# Novel retrieving device for coronary stent dislodgement

**DOI:** 10.1186/s12872-024-04377-x

**Published:** 2024-11-29

**Authors:** Zhan-Ying Han, Yong-Jian Zhu, Wen-Jie Lu, Zhi-Fang Wang, Jian-Feng Yang, Wen-Cai Zhang, Chun-Guang Qiu, Jian-Zeng Dong

**Affiliations:** 1 Department of Cardiology, The First Affiliated Hospital of Zhengzhou University, Zhengzhou, China; 2 Department of Cardiology, Xinxiang Central Hospital, Xinxiang, China; 3 Department of Cardiology, The Fifth Affiliated Hospital of Zhengzhou University, Zhengzhou, China

**Keywords:** Percutaneous coronary intervention, Stent dislodgement; retrieving device, Self-expanding basket

## Abstract

**Background:**

Stent dislodgement is a rare but particularly challenging complication. However, current treatment strategies are suboptimal.

**Objective:**

This study sought to preliminarily assess the feasibility of a novel self-expanding basket (SEB) catheter to successfully retrieve dislodged stent during percutaneous coronary intervention (PCI).

**Method:**

The novel SEB catheter is designed as a self-expanding basket tip made of superelastic shape nitinol memory alloy, which could automatically expand to tightly wrap and flatten the deformed struts regardless of whether the stent come off the guidewire. Consecutive patients with coronary artery disease who experienced stent dislodgement during PCI were included. The primary outcome was procedure success defined as completely removing the stent without surgical incision of blood vessels, or hemostatic forceps, or injury of access vessels.

**Results:**

From May 2020 to May 2023, a total of 6 patients encountering stent dislodgment were enrolled. Five presented as stent dislodgment with the guidewire in situ and the rest one as total stent and guidewire loss. Successful retrieving of dislodged stent with SEB catheter was achieved in 100% (6 of 6) subjects. After retracting lost stent, 5 of 6 patients received new stent implantation, and one only underwent balloon angioplasty with acceptable imaging results. No safety events were observed.

**Conclusions:**

This preliminary report of the novel stent retrieving device presents favorable efficacy and safety profile. Further multicenter study is required to confirm these findings.

**Clinical trial number:**

Not applicable.

**Supplementary Information:**

The online version contains supplementary material available at 10.1186/s12872-024-04377-x.

## Introduction

Starting from 1971, percutaneous coronary intervention (PCI) has undergone a serious of significant iterations, evolving from initial form of balloon angioplasty to subsequent bare metal stenting and further advancing to drug-eluting stents [1, 2]. As one of the most frequently medical interventions worldwide, PCI is currently recommended for the treatment of patients with acute coronary syndrome and those with chronic stable angina that unresponsive to optimal medical therapy [3, 4]. Despite advancements in stent design, adjunctive devices, procedure refinement, and potent antiplatelet medications enhancing the safety of PCI, the notable complications such as stent dislodgement continues to exist [5, 6].

Previous literature reported that stent dislodgement occurred from 0.32 to 8% during PCI, with substantially decreased incidence less < 1% since 2000 [7–9]. Extreme tortuosity, angulation, calcification and in-stent restenosis increase the risk of stent dislodgement from the delivery balloon [6]. Additional factors predisposing to the complication included inadequate predilatation, poor support of guiding catheter, and tip deformation caused by lesion or catheter [5, 6, 10]. The unintended stent dislodgement, although infrequent, can lead to severe systemic or intracoronary embolization. Currently, lost stents can either be retrieved, or deployed/crushed in the case of failed retraction. The available interventional methods of stent removal are small-balloon technique, double guidewire winding, as well as usage of retrieve trapped devices such as guiding extension catheter, snares, and biopsy forceps [11–13]. The main concern in the choice of the above techniques is the position of the guidewire. Indeed, some techniques, are not possible in the case of inadvertent removal of the guidewire [6].

In this proof-of-concept study, our aim is to preliminarily describe clinical experience with a self-expanding basket (SEB) catheter, which is novel retrieval device to successfully retrieve dislodged stent during PCI.

## Methods

### The device

**Fig. 1:**
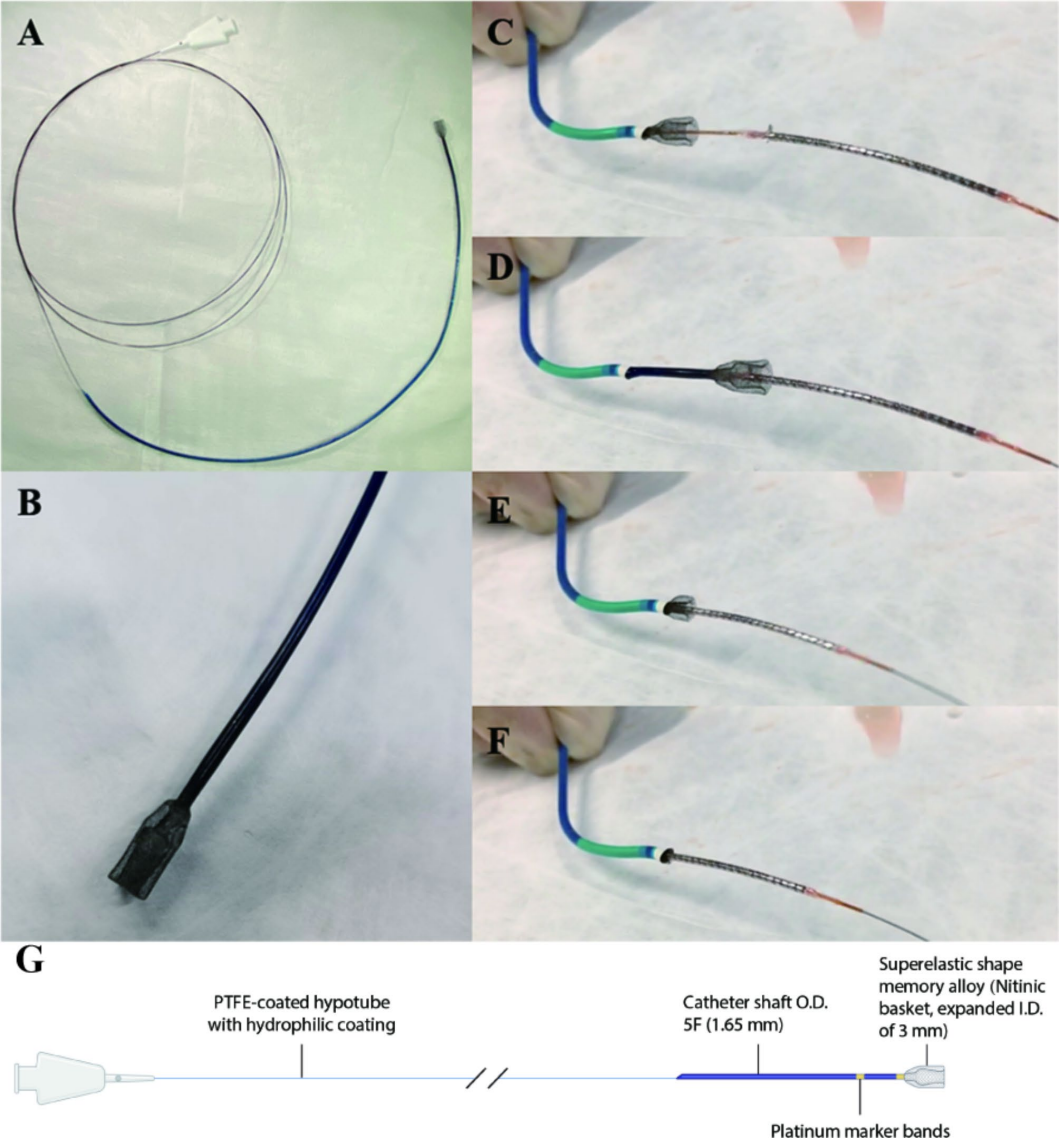
The SEB retrieving catheter device. The length of guide segment is 36-cm (outer profile of 1.65-mm, and inner diameter of 1.47-mm) (**A**). The distal tip is made of superelastic shape nitinol memory alloy (length of 12-mm, the maximum inner diameter after expansion of 3-mm) (**B**). Panels **C**-**F** shows the process of the device grasping the proximal deformed stent in vitro. (**G**) The detailed structure of SEB retrieving catheter device. SEB = self-expanding basket; O.D. = outer diameter; I.D. = inner diameter

The novel SEB catheter is made of a flexible, kink-resistant material, polyurethane, coated with a hydrophilic material on the outer surface, that allows it to navigate through intricate vascular pathways. Currently, the working length of the catheter is 150-cm, which consisting of proximal push rod, hypotube segment, guide segment (length of 36-cm, outer profile of 1.65-mm, and inner diameter of 1.47-mm) and distal tip. Especially, the distal tip was designed as a self-expanding basket (length of 12-mm, the maximum inner diameter after expansion of 3-mm) made of double-layer superelastic shape nitinol memory alloy (Fig. 1G and Video 1). The lumen of the basket is a continuation of the lumen of guide segment, allowing the passage of interventional devices such as wires, balloons, and stents. The distal basket can be easily compressed and passed through the lumen of a 6 F guiding catheter leaving about 50 µ m gap from the inner wall of the guiding catheter, which enable the optimal pushability, trackability and steerability. When the basket end is pushed out from guiding catheter, it will automatically expand to wrap the deformed struts, to avoid scratches between the detached stent and the guiding catheter (Fig. 1). More importantly, this device can work regardless of whether the stent come off the guidewire.

### Clinical cases

From May 2020 to May 2023, consecutive patients with coronary artery disease who experienced stent dislodgement during PCI and received SEB catheter treatment at the Department of Cardiology, the First Affiliated Hospital of Zhengzhou University were included. This study complies with the Helsinki Declaration and was approved by the Institutional Review Board of the First Affiliated Hospital of Zhengzhou University. The requirement for obtaining patient informed consent was waived because of the retrospective design. The clinical data of patients were collected through the electronic medical record system, comprising age, gender, diagnosis, and the characteristics of coronary lesions. The procedure information included access siting, targeted vessels, devices, and complications during hospitalization. The primary outcome was procedure success defined as completely removing the stent without surgical incision of blood vessels, or hemostatic forceps, or injury of access vessels.

### Procedure detail

According to the positioning of the balloon and wire during stent detachment, stent dislodgment can be classified into four types as following [14]: (1) Partial stent loss in the coronary artery (balloon partially within stent); (2) Total stent loss in the coronary artery with the guidewire in situ; (3) Total stent and guidewire loss in the coronary artery; (4) Coronary stent loss in the aorta or peripheral circulation. The SEB catheter could be applied to retrieve lost stent within large coronary artery (≥ 3 mm). The operation process of SEB catheter varies depending on whether the wire is completely lost.

**Fig. 2:**
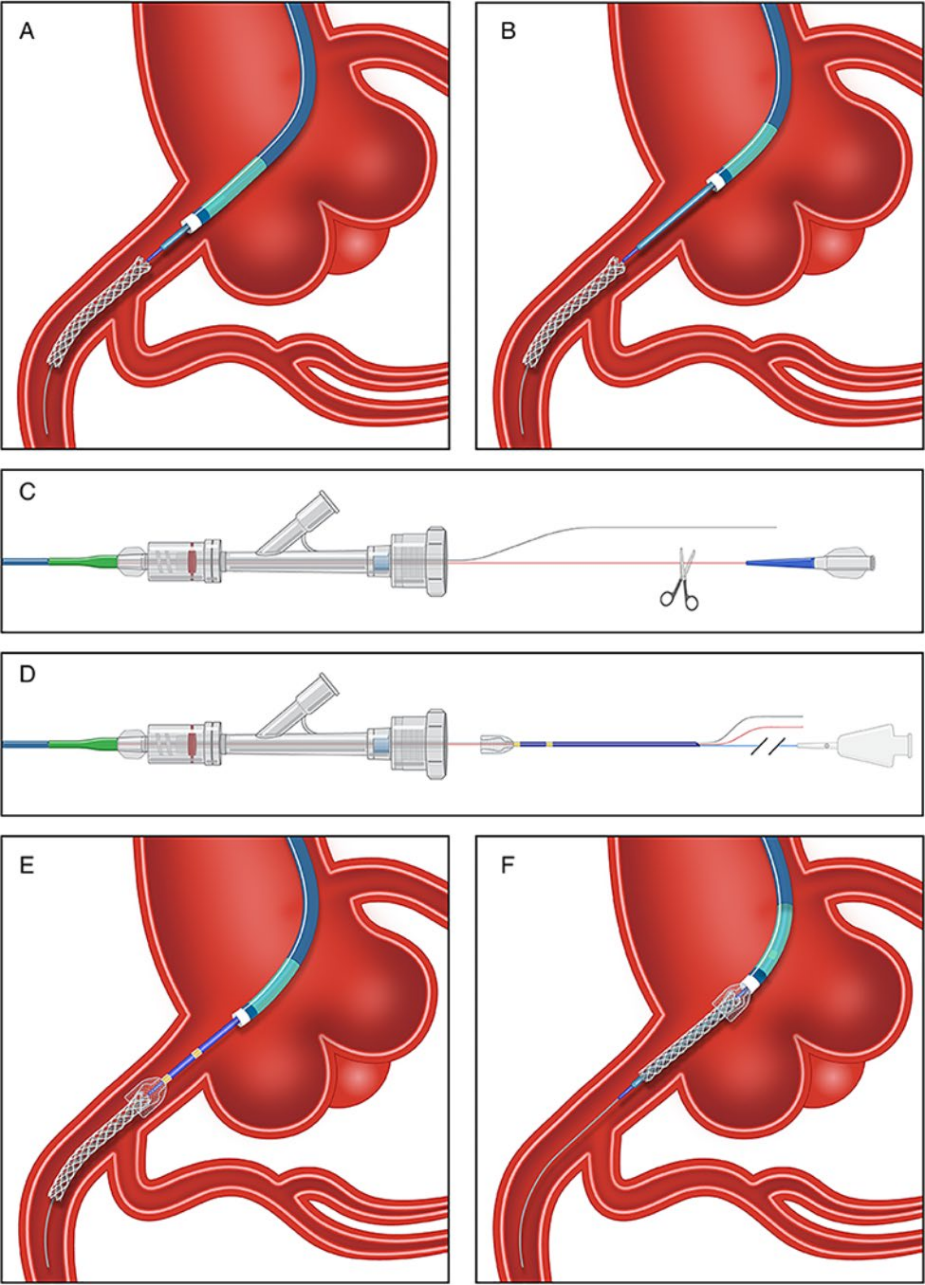
The Sketch of Total Stent Dislodgment with The Guidewire in situ. (**A**) Total stent detachment from loaded balloon. (**B**) Leaving at least 5 mm out of the ostial of guiding catheter to ensure that the head of the SEB catheter completely expand. (**C**) Trimming the distal hub of stent shaft due to the small inner diameter of distal basket and guide segment of the SEB catheter. (**D**) Advancing the SEB catheter through the track created by the stent shaft and guidewire. (**E**) Capturing the detached stent via pushing forward or withdrawing the basket. (**F**) Withdrawing the consortium of SEB catheter and stent. SEB = self-expanding basket

Partial or total stent dislodgment with the guidewire still in situ (Fig. 2, Supplemental Video 2): Firstly, the distal hub of stent shaft should be trimmed using a surgical blade or sterile scissors. This step is essential because the tail profile of stent shaft exceeds the inner diameter of distal basket and guide segment of the SEB catheter. Secondly, the SEB catheter could be sent into the guiding catheter through the track created by the stent shaft and guidewire, after compressing the distal basket end using a pre-loaded protective sheath. Thirdly, fully advancing the SEB catheter out of the ostial of guiding catheter at least 5 mm to ensure that the head of the SEB catheter completely expand in targeted vessels. And then, operator could capture the detached stent via carefully pushing forward or withdrawing the basket. Finally, the SEB catheter and stent could be simultaneously withdrawal to re-enter the guiding catheter, the consortium were tightly squeezed together due to compression from guiding catheter to prevent the stent from being lost again.

**Fig. 3:**
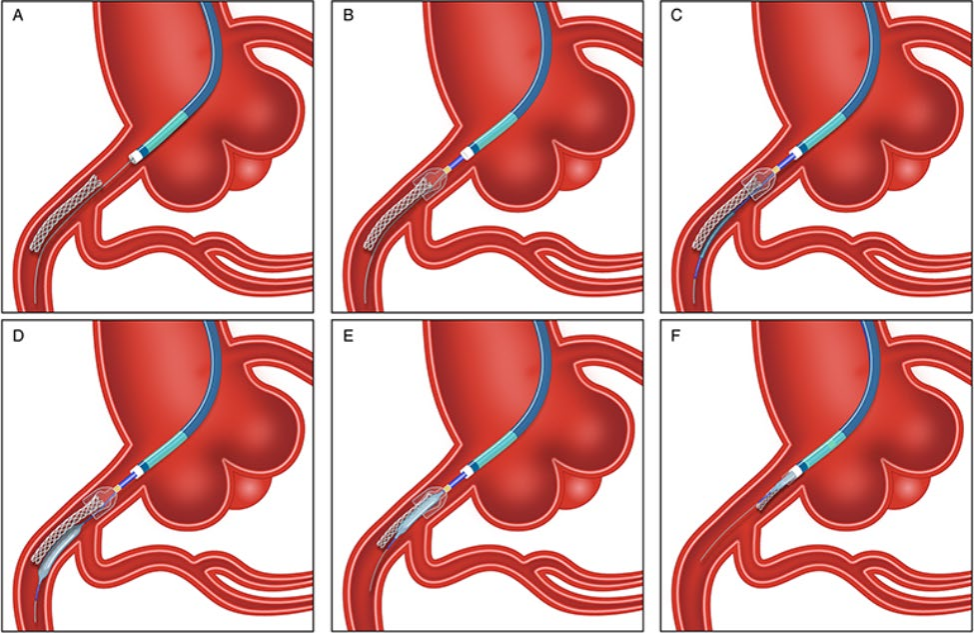
The Sketch of Total Stent and Guidewire Loss. (**A**) Sending a new guidewire parallel to the lost stent to the distal vessel. (**B**) Advancing the capture device to the proximity of stent via the new guidewire to trap the proximal stent. (**C**-**D**) Sending a small semicompliant balloon positioned the new guidewire to the location of stent dislodgement. (**E**-**F**) Under the support of low-pressure dilated balloon, retracting the consortium of retrieving device and lost stent into the guiding catheter

Total stent and guidewire loss (Fig. 3): We have reported the first-in-human case of successfully retrieving dislodged stent with total loss of guidewire and balloon [15]. In this scenario, it is unnecessary to retain the prior loaded guidewire and balloon in guiding catheter. Firstly, a new guidewire was sent parallel to the lost stent to the distal vessel. Secondly, the capture device was advanced to the proximity of stent via the new guidewire, and automatically expanded gauze basket to trap the proximal stent. Different from partial stent dislodgment, we suggest using a small balloon to assist in capturing stent from the perspective of increasing success rate and safety. In detail, a small semicompliant balloon positioned the guidewire was sent to the location of stent dislodgement. Under the support of low-pressure dilated balloon, the consortium of retrieving device and lost stent was completely retracted into the guiding catheter.

### Statistical analysis

The data of this pilot study are presented descriptively. No formal hypothesis testing or statistical comparisons were done. Continuous variables are presented as mean with standard deviation. Categorical variables are presented as absolute numbers and percentages.

## Results

### Clinical characteristics

A total of 6 patients with retrieval of dislodged stent using SEB catheter were included. Table 1 summarized the baseline clinical and procedure characteristics. Their age ranged from 59 to 70 years with equivalent gender distribution. All patients manifested as acute coronary syndrome and multivessel diseases. The majority of *de novo* targeted vessels located in right coronary artery (RCA) (5/6, 83%), leaving one in LCX. Tortuosity and angulation could be identified in 5 target lesions, and calcified features existed in 4 target lesions.

**Table 1:** Baseline characteristics of patients

Cases	Sex	Age	Vessel	Access	Guiding catheter	Semi-compliant balloon (mm)	The maximum diameter of pre-dilatation balloon (mm)	Type	Procedure success	Time^*^ (min)	Final stent implantation	Complication
1	Female	68ys	RCA	RR	6 F SAL 0.75	2.0 × 20	2.5 × 13 (NSE balloon)	Partial stent dislodgment with the guidewire in situ	Yes	10	Yes	No
2	Male	65ys	LCX	RR	6 F EBU 3.75	1.5 × 20	/	Total Stent and Guidewire Loss	Yes	6	No^#^	No
3	Female	70ys	RCA	RR	6 F SAL 1.0	2.0 × 20	/	Partial stent dislodgment with the guidewire in situ	Yes	10	Yes	No
4	Male	59ys	RCA	RR	6 F SAL 0.75	2.0 × 20	2.5 × 15 (Non-compliant balloon)	Total stent dislodgment with the guidewire in situ	Yes	12	Yes	No
5	Female	67ys	RCA	RR	6 F SAL 0.75	2.0 × 20	3.0 × 12 (Non-compliant balloon)	Total stent dislodgment with the guidewire in situ	Yes	10	Yes	No
6	Male	66ys	RCA	RR	6 F JR 0.75	2.0 × 20	/	Partial stent dislodgment with the guidewire in situ	Yes	8	Yes	No

^*^Time from initiating retrieving catheter to successfully retracting stent. ^#^Only balloon angioplasty with satisfied results. RCA = right coronary artery. LCX = left circumflex coronary artery. RR = right radial artery

### Clinical cases

**Fig. 4:**
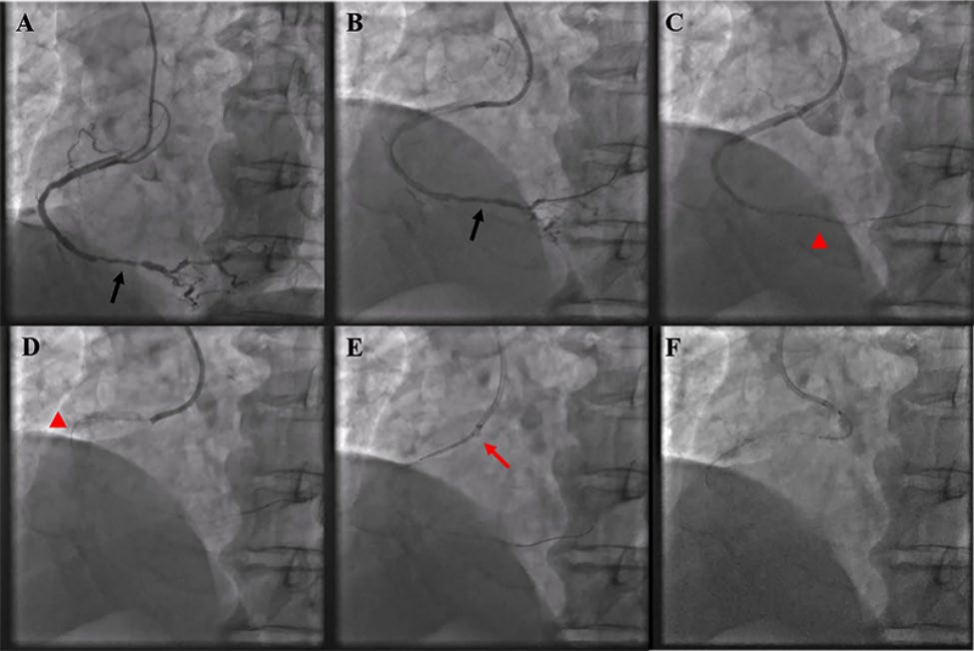
The Clinical Scenario of Total Stent Dislodgment with the Guidewire in situ. (**A**) Diffuse severe stenosis in distal right coronary artery (black arrow) and prior implanted stent in proximal vessel. (**B**) After predilation (black arrow). (**C**) Failing to send stent crossing the lesion (red arrowhead). (**D**) Lost stent in proximal stent struts (red arrowhead). (**E**) Advancing the SEB catheter through the track created by the trimmed stent shaft and guidewire and catching the deformed lost stent (red arrow). (**F**) Retracting the device and lost stent into the guiding catheter

**Fig. 5:**
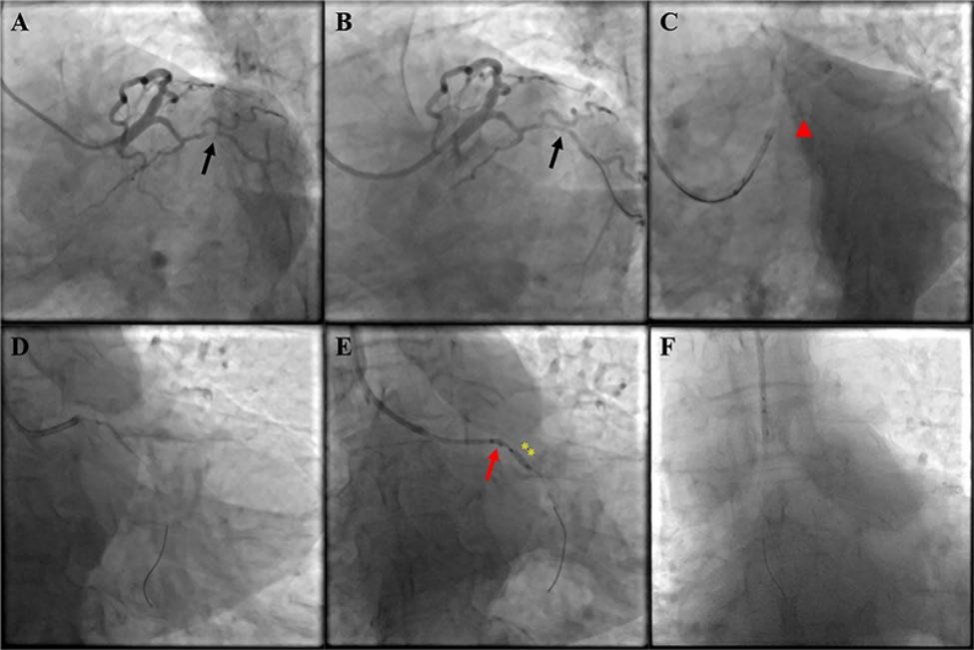
The Clinical Scenario of Total Stent and Guidewire Loss. (**A**) Subtotal occlusion with serious tortuosity and calcification in the middle of the left circumflex coronary artery (black arrow). (**B**) After predilation (black arrow). (**C**) Failing to send stent crossing the lesion (red arrowhead). (**D**) Lost stent and guidewire (red arrowhead). (**E**) Advancing the SEB catheter through a new guidewire to grasp the deformed lost stent with the support of low-pressure dilated balloon (yellow asterisks). (**F**) Retracting the device and lost stent into the guiding catheter

In the initial strategy, adequate lesions preparation using semicompliant balloon or noncompliant balloon were completed. The details of devices usage were presented in Table 1. Against this background, pre-specified stents failed to be advanced through the target lesions and were lost in proximal coronary arteries. Five presented as stent dislodgment with the guidewire and/or balloon in situ and the remaining one as total stent and guidewire loss. Successful retrieving of dislodged stent with SEB catheter was achieved in 100% (6 of 6) subjects. The average time from initiating the process to successfully removing the stents is 9.3 ± 2.1 min. After retracting lost stent, 5 of 6 patients received new stent implantation, and one only underwent balloon angioplasty with acceptable imaging results. We herein illustrated two representative cases (Figs. 4 and 5) to clarify the overall workflow of SEB catheter.

### Follow up

Patients were discharged from the hospital in good condition, without severe periprocedure complications including death, myocardial infarction, coronary dissection, stent embolization, and injury of access vascular. All patients are doing well to date (follow-up of up to 36 months postprocedure, range 5–36 months).

## Discussion

Herein, we report the clinical cases with a novel self-expanding basket catheter to retrieve dislodged stent during PCI. The initial experience exhibits excellent procedural efficacy and safety. Success retrieval of lost stent are achieved in all patients with no in-hospital or long-term adverse events. The dedicated catheter is also easily manipulated and retrieved, and can be used in various types of stent dislodgment.

Stent dislodgement is a rare but particularly challenging complication during PCI. Although low incidence, this complication may cause serious consequences, significantly increasing the incidence of perioperative complications, and improper management may even lead to death [6, 9, 16]. The key point to prevent stent detachment for interventional cardiologists is to predict and identify high-risk lesions characterized as angulation, calcification, and diffuse lesions, as well as the proximal implanted stent [11]. Insufficient predilation and non-coaxial guiding catheter commonly exacerbate the difficulties in advancing stent, thereafter improper retracting could cause stent deformation and detached from loaded balloon, ultimately embedded in lesions or prior stent. European scholars divided stent dislodgement into four types according to whether the balloon or wire within stent [14]. Until now, there are no straightforward techniques applicable to all situations. Some interventional techniques have been utilized to successfully retrieve lost stent in reported cases, including small balloon technology, gooseneck snares, catheter extension technology, and even biopsy forceps [12, 17, 18]. However, the current interventional approaches are not always appropriate or possible in retrieving stent in all cases [19]. Moreover, technical difficulties and potential injury in coronary artery and access vessels are also concerned issues.

The novel equipment is suitable for various types of dislodged stent. Some tricks and tips should be noted for different clinical scenarios. In the cases of guidewire within the lost stent, the essential step is to trim the distal hub of stent shaft. And the SEB catheter could be sent through the track created by the stent shaft and the loaded guidewire. For patients without guidewire within in the lost stent, the SEB catheter can be directly advanced to the proximal of stent via a new guidewire, which only needs to be sent to the distal vessel without reentering the stent again. The operation method of the novel SEB catheter is similar to existing interventional instruments like guide-extension catheter or snare, hence shortening the learning curve. Even though, it should be noted that potential coronary damage is possible for less-experienced operators, especially in relatively small blood vessels. Another risk is the possibility of pushing the lost stent towards the distal end of the coronary artery when inappropriate procedures are performed. Complex lesions such as tortuosity or calcification can also increase the probability of device failure.

Compared with the other available intracoronary foreign body retrieving devices, the SEB system has some unique characteristics. Different from Dormia basket [20], our device provides a larger contact area and denser steel beam, ensuring tightly grasping the stent and reducing the possibility of pushing the stent towards the distal vessel. The nitinol gooseneck snare has been widely applied to the cases of misplaced coronary stent [6, 18, 21]. The snare technique often requires large access vessels (e.g., femoral artery), and the primary procedure difficulty is inability to advance the device around the object. In some complex cases, procedure failure of gooseneck snare is not uncommon despite of multiple attempts [22]. Due to the design of scalable gauze basket end, the current device could automatically expand to fully fit the coronary arteries diameter, and easily to grasp the of the deformed proximal stent steel beam; During the retreating, the soft basket could be contracted to further tighten the stent to increase stability, and they are gradually inserted into the guide catheter, and then removed from the body.

Although the endovascular approach is still the first-line choice for retrieving dislodged coronary stent, a multidisciplinary decision of the optimal plan is mandatory to approach these challenging situations [12]. Interventional cardiologists should be well-trained to master skills in endovascular procedures and have good knowledge of the anatomy. More importantly, one must maintain flexibility to modify the initial strategy when encountering different unexpected situations [11]. Balancing the risks and benefits is significant because repeated retrieval attempts may cause coronary artery injury, as well as disastrous consequences [23]. If unsuccessful attempts for retrieval, it may be appropriate to apply stent crushing to a suitable vascular bed or deployment of another stent alongside the lost one [5, 6]. Finally, open surgery retrieval is still indicated in some cases [19, 24].

### Limitations

This proof-of-concept study was designed to assess the feasibility and safety of the retrieving system and unable to provide clear evidence of benefits. Limited by the small sample size and the single-arm treatment, the study findings should be considered as hypothesis-generating. A head-to-head comparison with other techniques in the future is necessary to validate the efficacy and safety of this device. Considering highly selective patients and experienced experts in the current study, the generalization of our findings should be cautious. Additionally, the ideal indication of this device is stent lost in large vessels. The capture equipment cannot be used for distal vessels that are difficult to reach.

## Conclusion

The current evaluation shows the feasibility of the dedicated stent retrieving system. The device successfully retrieved dislodged stent in all cases and no vascular injury was observed. Further large sample size study is needed to evaluate device safety and potential complications.

## Electronic supplementary material

Below is the link to the electronic supplementary material.

Supplementary Material 1

Supplementary Material 2

## Data Availability

The data that support the findings of this study are available from the corresponding author upon reasonable request.
